# Voice‐Assisted Technology as a Potential Tool for Addressing Speech and Voice Concerns Experienced by People With Parkinson's Disease and Other Conditions Presenting With Dysarthria: A Scoping Review

**DOI:** 10.1111/1460-6984.70140

**Published:** 2025-10-20

**Authors:** Jodie Mills, Orla Duffy, Katy Pedlow, W. George Kernohan

**Affiliations:** ^1^ Speech and Language Therapy School of Health Sciences Ulster University Belfast UK; ^2^ Physiotherapy School of Health Sciences Ulster University Belfast UK; ^3^ Institute of Nursing and Health Research, Ulster University Belfast UK

**Keywords:** dysarthria, Parkinson's disease, voice‐assisted technology

## Abstract

**Background:**

People with neurological conditions such as Parkinson's Disease are at risk of speech and voice difficulties that impact volume, clarity of speech and intelligibility. Voice‐assisted technology (VAT), such as Alexa, poorly recognises speech difficulties, and this often prompts people to change their speech to enable interaction.

**Aims:**

This study aims to identify and map available literature regarding the utilisation of VAT to address speech and voice difficulties associated with neurological conditions. We explore (1) how well VAT recognises speech difficulties or dysarthric speech, across different severities and intelligibilities, and (2) the impact of using VAT on intelligibility, clarity or volume for people with dysarthria or speech difficulties.

**Methods and Procedures:**

A review of available literature was guided by Arksey and O'Malley's scoping review framework between September 2023 and November 2023. Five electronic databases were systematically searched, yielding 840 results. Results were screened by title, abstract and full text using inclusion and exclusion criteria. Eligible papers were identified and articles using ‘out of the box’ (i.e., not customised) VAT, speech difficulties or dysarthria and speech outcomes were included. Relevant data were extracted using an adapted version of the Joanna Briggs Institute extraction tool, and study outcomes were narratively reviewed. We report findings using the Preferred Reporting Items for Systematic Reviews and Meta‐analysis extension for scoping reviews (PRISMA‐ScR).

**Outcomes and Results:**

Five papers were reviewed that included varying speech difficulties, including dysarthria, stammering and reduced intelligibility. Results yielded limited evidence regarding the role of VAT for assessment in speech and language therapy (SLT). However, literature suggests that VAT should be used cautiously as a tool for managing volume, intelligibility and clarity of speech. Indeed, implications for activity, participation and well‐being were noted, although findings are mixed. Early outcomes indicate that further research is needed to validate the evidence base for using VAT in SLT. Future research should explore the views of people with Parkinson's and speech and language therapists (SaLTs) who are using VAT to understand current usage and direct future development.

**Conclusions and Implications:**

This review provides tentative conclusions that VAT may contribute to improve volume, intelligibility and clarity of speech for people with dysarthria or speech difficulties, such as people with Parkinson's Disease. However, caution is advised when using VAT with dysarthric ethnic speakers, as word error rates of devices are high, and it is unclear if this is related to device limitations or disordered speech. Biofeedback from devices may contribute to the mechanism of the effect.

**WHAT THIS PAPER ADDS:**

*What is already known on this subject*

- Voice‐assisted technology poorly and inconsistently recognises dysarthric or disordered speech, often without a clear indication of whether poor recognition is a limitation of the technology itself or the result of dysarthric or disordered speech.

*What this paper adds to existing knowledge*

- Voice‐assisted technology may hold promise as a future SLT management tool to improve volume, clarity and intelligibility of speech, with implications for activity, participation and well‐being. Feedback from devices and the enablement of home practice may contribute to the technology's mechanism of effect; however, device limitations in recognition rates, variability and transactional interactions must also be considered. Due to a lack of evidence‐based intervention development, no firm recommendations can be made on the specific impact for different populations, on dosage or duration of use during interventions, or on the usage of voice‐assisted technology in assessment. Additional research is needed to fully understand clinical implications.

*What are the potential or actual clinical implications of this work?*

- Voice‐assisted technology may be used to promote changes in voice and speech, including improved volume, intelligibility and clarity of speech and potential functional gains.
- However, considering the numerous variables that can influence the consistency of VAT, clinicians should undertake a thorough risk–benefit analysis and determine its appropriateness on a case‐by‐case basis, in light of the potential adverse outcomes, whether real or perceived. People living with Parkinson's may benefit from using VAT as part of SLT. Devices may allow unlimited practice attempts, can prompt increased self‐awareness of speech difficulties and motivate continued practice. However, error rates, ethnocentric bias and potential impacts on well‐being warrant caution and clinicians should use clinical judgement to carefully consider clinical use of VAT. Overall, this widely available technology has potential as an SLT tool to enable self‐management and support maintenance, alongside or following therapy. Future research should seek to investigate biofeedback as a mechanism of speech change.

## 1 Introduction

### 1.1 Parkinson's Disease

The Global Burden of Diseases, Injuries and Risk Factors Study reported that the prevalence of Parkinson's Disease has more than doubled from 2.5 million in 1990 to 6.1 million in 2016 (Dorsey et al. 2018). An 18% increase is predicted by 2025 (Parkinson's U.K. 2017).

One of the complex challenges faced by people with Parkinson's Disease is loss of spoken communication. Most people (90%) with Parkinson's Disease (PwPD) present with voice changes, 45% with articulation difficulties and 20% with fluency disorders (Miller et al. 2011; Ho et al. 1999). Deterioration in neuromuscular control in Parkinson's Disease contributes to hypokinetic dysarthria, defined by reduced loudness, and intelligibility, imprecise articulation and a harsh breathy quality (Miller 2017; Darley et al. 1969). PwPD also have impaired loudness perception levels (De Keyser et al. 2016; Contreras‐Ruston et al. 2024), which creates difficulties producing the correct volume, and many people feel they are shouting when speaking at normal volume. Progressive dysarthria can impact intelligibility, significantly reducing participation in activities, affecting quality of life and self‐identity (Miller and Walshe 2011).

Lee Silverman Voice Treatment (LSVT) LOUD is regarded as gold standard for treating dysarthria secondary to Parkinson's Disease (Fox et al. 2012; Ramig et al. 2018). However, access to speech and language therapy (SLT) is limited for PwPD, and therapists are unable to meet recommendations for early interventions (Miller et al. 2011). This may be due to the resource‐intensive nature of LSVT LOUD for clinicians and staffing shortages, as well as the high level of commitment required from service users. Although LSVT LOUD is a personalised intervention programmed to promote maintenance and generalization of gains, significant time and effort must be given to home practice post‐therapy. These high‐effort exercises reportedly reduce motivation to complete home practice (Yorkston et al. 2017) and were not sufficient to remove barriers to communicative aspects of participation (Baylor et al. 2023). This contributes to difficulties maintaining the long‐term outcomes reported in research (Ramig et al. 2018) and indicates that alternatives to promote therapeutic gains, generalisation and maintenance should be explored, including a better understanding of the role of technology to facilitate SLT.

### 1.2 Voice‐Assisted Technology (VAT)

Technology‐based interventions may act as a home therapy. This could afford PwPD increased home‐practice opportunities for SLT and highlight relevance to SLT clinical practice. Given one in five homes across the United Kingdom have a smart speaker (Centre for Data Ethics Innovation 2019), Voice‐Assisted Technology (VAT) is an everyday device, which offers an accessible option. This review discusses VAT as a therapeutic tool to rehabilitate impaired speech and voice difficulties and the potential voice‐related impacts on activity, participation and well‐being as a result of therapeutic usage, in line with the International Classification of Functioning (ICF) framework.

VAT is defined as a device which uses natural language processing or Automatic Speech Recognition (ASR) to interpret words and convert them into actionable requests. In this review, VAT refers to commercially available or accessible VAT, for example, Google Cloud, Amazon Alexa or Google Home, the latter two having been available since 2014 and 2017, respectively.

These commercially available, off‐the‐shelf devices are everyday technologies that are not primarily developed or intended for clinical use, but could be implemented by speech and language therapists (SaLTs) in clinical practice. This provides a rationale for our definition and latter inclusion criteria outlined in Table 1. This rationalises the focus of the review: establishing the current evidence for rehabilitative effects of VAT on speech and voice outcomes. Non‐commercial devices are described as technologies, which are not off‐the‐shelf devices, and are specifically designed to improve ASR or are created for use in SLT. These are not considered to be everyday technologies. Much of the wider literature indicates the potential of VAT as an environmental accessibility tool. While this is acknowledged, it is not the intention of this paper to examine VAT as an environmental controller. Here, literature is explored to establish if there is a prima facie therapeutic benefit of VAT usage.

**TABLE 1:** Inclusion/Exclusion criteria used to guide abstract and full‐text screening in the review.

Inclusion criteria	Exclusion criteria
Empirical research regarding VAT recognition of dysarthria speech and speech difficulties and promotion of speech change	Research which examines VAT as an environmental controller or as an accessibility tool to help PwPD perform tasks
Meets the definition of VAT and includes a form of feedback to the user	Games and non‐standard apps
Out of the box use of VAT with no modifications to technology (a commercial, readily available everyday technology)	Studies which: adapt VAT or create/code new applications to improve the technology's automatic speech recognition of dysarthric speech, develop technology specifically for speech and language therapy usage (devices considered non‐commercial as they are not an everyday technology)
Empirical research, conference proceedings, theses and dissertations	Literature reviews, systematic and scoping reviews and studies that do not present original data
Full text available in English	
Adults aged 18+ with a speech or voice difficulty associated with Parkinson's (dysarthria or voice problems or stammering)	Aphasia Dementia Other cognitive communication disorders (where the research focus is on language/disrupted language use)
Papers from 2014 onwards (given that the first commercially available VAT, Amazon Alexa was developed in 2014 and this commercial technology may be readily implemented in clinical practice)	

Despite perceptions that older people are resistant to technology, Kowalski et al. (2019) highlighted a high level of smart device acceptance, with positive reflections regarding the non‐judgmental, paced interactions with VAT. As such, VAT may offer a potential domestic solution for speech difficulties, allowing therapeutic gains through practice with the VAT device. While this review focusses on the therapeutic potential of VAT in relation to impaired speech and voice difficulties, it also examines voice‐related impacts for activity, participation and well‐being potentially arising from VAT use.

### 1.3 VAT and Speech Difficulties

Several literature reviews have suggested that VAT poorly recognises dysarthric speech (Young and Mihailidis 2010; Mustafa et al. 2015; Jaddoh et al. 2023). Recognition may be impacted by reduced volume, variable articulation and severity of speech difficulties (Young and Mihailidis 2010; Mustafa et al. 2015). This may be because phonemes are less clearly defined, making it difficult for speech to be converted accurately into text for further processing.

The potential role of VAT in SLT therapeutic practice has been highlighted by research. It has been suggested that poor recognition of dysarthric speech may allow VAT to provide feedback on volume, clarity and intelligibility of speech (Kulkarni et al. 2022). This prompts participants with speech difficulties to change their speech to enable interaction with VAT, and the everyday nature of devices and use in the general population can de‐medicalise therapy and speech practice. VAT provides objective and unjudgmental biofeedback on speech clarity and volume (Kulkarni et al. 2022), which results in speech adaptations by users and can promote self‐monitoring and increased self‐awareness by recalibrating speech feedback loops. For example, Pradhan et al. (2018) conducted a secondary review of Amazon Alexa reviews, and 13.6% described interactions with a speech difficulty, and found 2% of reviews highlighted VAT made them speak ‘slowly, clearly and loudly’. This is echoed by McNaney et al. (2020) and Denman and Jones (2019), who demonstrated that dysarthric young people were rated as more intelligible when speaking a target sentence to a smart speaker. This highlights the potential of VAT to promote therapeutic changes in speech and voice; however, a review of currently available evidence is required to establish if there is a beneficial effect of VAT use on speech and voice outcomes. While it is acknowledged that people with dysarthria and other speech impairments may use VAT as it has several enjoyable and useful features, this review discusses the impacts of VAT on impaired speech and voice, and speech and voice‐related impacts on activity, participation and well‐being. For example, uses of VAT for environmental control or entertainment are considered to relate to accessibility rather than the therapeutic change of speech and voice and are therefore excluded from discussion in this review

Research indicates that improvements in speech intelligibility or voice are often a secondary outcome of usage, rather than the main factor for adoption of VAT (Smith et al. 2021). A recent scoping review by Cave and Bloch (2023) explored how ASR technology is used by people with amyotrophic lateral sclerosis (ALS). However, researchers did not investigate the therapeutic value of VAT to change or improve speech outcomes with this population or explore the usefulness of ASR across neuro‐degenerative disease progression, instead investigating the technology as a speech recognition tool. This highlights the need to explore how VAT usage may impact speech and voice outcomes from a therapeutic perspective. Findings highlighted that feedback from ASR technologies, such as that used within VAT, may be used to monitor neuro‐degeneration in speech in a therapeutic way, thus indicating the therapeutic potential of VAT. Therefore, this review seeks to build on this foundation and extend it to explore the therapeutic applications of VAT as a SLT tool and explore potential beneficial effects of usage on speech and voice outcomes.

Overall, there is emerging evidence that VAT can promote positive speech changes for people with dysarthria or speech difficulties and a rationale to support why people with speech and voice difficulties themselves would use VAT. No reviews to date have explored the therapeutic potential for VAT in an SLT context, as a tool to support SLT outcomes or demonstrated clinically meaningful outcomes for people with dysarthria. Therefore, it is necessary to review the nature and extent of the literature surrounding the use of VAT in relation to speech outcomes in order to inform evidence‐based practice. As such, this review seeks to establish if there is a beneficial effect of VAT usage on speech and voice outcomes.

### 1.4 Aims and Objectives

The overall aim of this scoping review is to identify and map the available literature regarding the therapeutic utility of VAT for speech and voice difficulties, which display clinical similarities to Parkinson's Disease. A scoping review was deemed appropriate, as VAT is an emerging area, and it is necessary to scope the literature to identify and map the available evidence. One research question was addressed a follows:

What is known from existing literature about the utilisation of VAT for speech and voice difficulties associated with Parkinson's Disease and other conditions with related speech or voice characteristics? We report and discuss the following: (1) recognition rate of VAT, associated with severity and intelligibility, for users with dysarthria or a speech difficulty, and (2) improvements in speech or voice to include intelligibility, clarity and volume as a result of using VAT.

## 2 Methods

This scoping review follows the five steps outlined by Arksey and O'Malley (2005), further developed by Levac et al. (2010), with reference to the Joanna Briggs Institute (JBI) scoping review guide. The Preferred Reporting Items for Systematic Reviews and Meta‐Analysis Extension for Scoping Reviews (PRISMA‐ScR) (Tricco et al. 2018) was used as a guide for reporting the review and informs the rigour and credibility of findings. The scoping review protocol was registered on the Open Science Framework.

### 2.1 Search Strategy

Comprehensive literature searches were developed and conducted, using CINAHL Ultimate, Linguistic and Language Behavioural Abstracts (LLBA), Scopus, MEDLINE (Ovid) and ACM digital library, with support from a research librarian, and by using the Population, Exposure and Outcome framework. Keywords and Medical Subject Headings (MeSH), relating to VAT, speech difficulties and speech outcomes, were used. The final search strategy and terms are provided in Supporting Material 1. Searches were undertaken in October and November 2023, with the last search completed on 1st November 2023, retrieving 751 results. Searches were re‐run in June 2024, and while the number of search results increased to 840, no additional papers fitting the inclusion criteria were noted.

### 2.2 Selection of Sources of Evidence

#### 2.2.1 Study Selection

Search results were imported into literature review software (Covidence), and reference lists were also hand searched to augment database searches (Peterson et al. 2017). Five percent of the results titles and abstracts were independently assessed by all four of the research team to pilot the eligibility criteria (see Table 1) and to ensure inter‐rater agreement and consistency (Peters et al. 2020).

**FIGURE 1:**
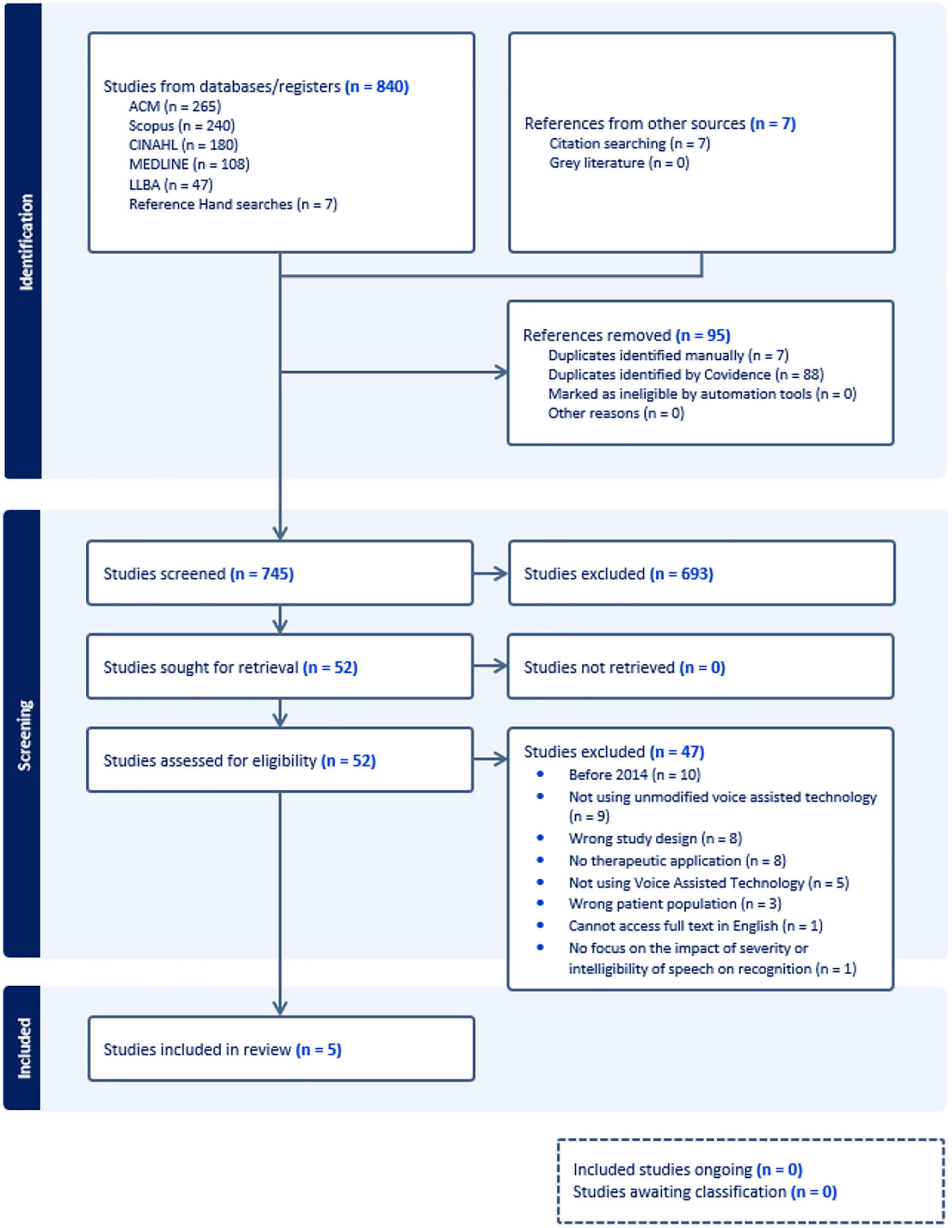
Showing the search, screening, identification and inclusion process of the current scoping review.

The final list of titles and abstracts was screened independently by researchers (J.M., O.D., K.P., W.G.K.) to assess eligibility for inclusion, followed by full‐text screening. Any discrepancies were resolved through discussion and consensus. Those who met certain inclusion and exclusion criteria were progressed for data extraction. Figure 1 presents a summary of the process.

#### 2.2.2 Data Charting and Reporting the Results

The data extraction and charting framework (Supporting Material 2) was adapted from Cave and Bloch (2023), and the JBI framework (Peters et al. 2020) to suit the needs of the current review (Levac et al. 2010). This included authors, year of publication, title, aims and objectives, overview of study characteristics, participant details and communication needs, technology used, execution of delivery, duration and dosage of intervention, purpose of VAT use, outcome measures, findings and researcher‐reported limitations. Data were extracted onto a spreadsheet (Microsoft Excel) and was piloted by the research team (J.M., O.D., K.P., W.G.K.) on two of the papers, to enhance reliability and prevent bias. The data extraction table and results were also further checked by the authors.

## 3 Results

### 3.1 Search Results

The searches yielded 840 articles, and 745 remained following removal of duplicates. Following the title and abstract screening, 52 studies were included for full‐text review, and five papers were included in the present review (Figure 1). Reasons for exclusion included: date before 2014 (*n* = 10), not using VAT (*n* = 5) or out of the box technology (*n* = 9), not having therapeutic application (*n* = 8) and wrong study design (*n* = 8), patient population (*n* = 3) or no speech and voice difficulties present (*n* = 2), inability to access the full text in English (*n* = 1) and no focus on the impact of severity or intelligibility on speech recognition (*n* = 1). The characteristics (Table 2) and findings of included studies are presented (see Table 3).

**TABLE 2:** Showing the characteristics of the five included studies.

Name of publication	Exploring Smart Speaker User Experience for People Who Stammer	Attitudes towards the use of voice‐assisted technologies amongst people with Parkinson's Disease: Findings from a web‐based survey	Validity of Off‐the‐Shelf Automatic Speech Recognition for Assessing Speech Intelligibility and Speech Severity in Speakers With Amyotrophic Lateral Sclerosis	Speech and Language Practitioners’ Experiences of Commercially Available Voice‐Assisted Technology: Web‐Based Survey Study	Smart Speaker Devices Can Improve Speech Intelligibility in Adults With Intellectual Disability
Authors	Bleakley et al.	Duffy et al.	Gutz et al.	Kulkarni et al.	Smith et al.
Year of publication	2022	2021	2022	2022	2021
Study location	Ireland	United Kingdom	United States	United Kingdom	United Kingdom
Population	People with a stammer	People with Parkinson's	People with ALS	Speech and language therapists	People with intellectual disabilities
Time since diagnosis/disease severity	Severity not assessed	66.9% reported they had experienced speech changes associated with Parkinson's	Mean ALSFRS‐R score was 31—Mild to moderate disease progression	Not applicable	Mild to moderate intellectual disability
Methodology	Qualitative—Diary entries and semi‐structured interviews	Online mixed methods survey	Non‐randomized experimental design	Online mixed methods survey	Semi‐randomized control trial
Sample size	*N* = 11	*N* = 290	*N* = 72	*N* = 230	*N* = 48
Main purpose of VAT use	Speech change	Speech change	Speech recognition	Speech change	Speech change
Technology used	Google Home	Commercial out of the box VAT	Google API	SaLTs who had used VAT used Alexa, Siri and Google Assistant	Amazon and Google commercial out of the box VAT
Outcome measures	Narrative self‐report of experiences	Self‐reported changes in speech linked to VAT	Intelligibility ratings (VAT vs. human speech and language therapist (SaLT) rate, reflective of sentence‐level speech intelligibility) and severity ratings (VAT vs. human SaLT recognition rate)	Self‐reported impacts, usage and perspectives	Intelligibility ratings by 24 blinded SaLT students but were more consistent with speech clarity as raters were shown the target word or phrase during ratings
Research limitations	Unable to analyse device transcription of speech due to GDPR. Did not quantitively measure changes on speech as a result of using VAT.	Did not quantitatively measure changes in speech because of using VAT. Electronic survey may self‐select PwPD actively engaging with technology and those who have used VAT.	Findings are cross‐sectional so cannot be applied to progress monitoring. Research examined sentence‐level intelligibility and ASR may perform differently at the word, phrase and discourse level.	The survey sample size was not large enough to obtain 90% confidence levels and was web‐based, self‐selecting users of technology and VAT. Did not state if participants were users of VAT or had been introduced via SLT.	Study was limited to a 12‐week duration, so uncertainty about gains from longitudinal use and moderate effect size was not compared to effects from traditional SLT. Unable to measure dose‐dependent effects or the impact of types of utterance due to GDPR.

**TABLE 3:** Showing findings from the five papers reviewed, highlighting direct speech changes and therapeutic applications.

Title	Bleakley et al. (2022)	Duffy et al. (2021)	Gutz et al. (2022)	Kulkarni et al. (2022)	Smith et al. (2021)
**Key findings**					
How well is speech recognized	Not reported	43% of PwPD with voice difficulties said it works most of the time, 31% said it works some of the time, 37% said VAT understands them most of the time, although they have to repeat sometimes, and it never works for 3.0%. Recognition impacted by unclear speech, dysfluency, low volume and accents.	ASR recognition rates: 82% normal intelligibility, 77% mild, 49% moderate, 24% severe and 3.9% profoundly impaired intelligibility, indicating a measurement floor. ASR did not differentiate between normal and mild dysarthria, only classifying—normal, severe and profound speech.	Recognition impacted by accents, unclear speech, low volume speech SaLTs who had used VAT found high success rates with clients with severe speech impairments.	Not reported
Direct speech changes	Not reported	42% reported no change, 25% were asked to repeat less by VAT, 15% had a clearer voice, 14% increased confidence in their speech, 13% had a louder voice, 10% were asked to repeat less by others, and 9.3% decreased speech confidence.	Not reported	53% (120/230) believed VAT could help their clients speak louder, 47% (108/230) believed it could help clarity of speech, 10% (18/175) reported increased intelligibility, and may allow voice maintenance.	Greater increase in intelligibility ratings related to device use for the intervention group using VAT. Slightly smaller effect for unrelated words.
Strategies to adapt speech	Increasing volume, getting closer to the speaker, speaking slowly, over‐articulating, repetition of commands, diaphragmatic breathing and planning utterances.	Increasing volume, speaking clearly, increasing clarity of speech/over‐articulating, decreasing rate of speech.	Not reported	Increasing volume, speaking slowly, over‐articulating/speaking clearly.	Not reported
VAT as a therapeutic tool	VAT could be used for professional speech and language therapy, for example, opportunities for home practice following SLT or informed by SaLTs to complement therapy.	Practice tool for volume levels by encouraging self‐awareness.	Poor early indicator of speech deterioration and not appropriate for outcome measurement, evaluating progression of speech severity or tracking precise speech changes. Works for broad detection of severe dysarthria. Limited utility for mild/profound and uncertainty for moderate.	Practice tool in clinic to support explicit training of SLT strategies, outcome measurement to support home‐based therapy programs, continuous device feedback, provides feedback on speech clarity and improving self‐awareness.	Home practice encourages repetition and motivation to complete exercises, device feedback on clarity of speech and may facilitate spaced repetition and positively reinforce behaviours.
Advantages of VAT	Build confidence by practicing words/strategies, lack of perceived judgement, reduced anxiety and improved fluency and increased awareness of fluency difficulties	Speech to text and voice functions helped overcome physical limitations, can be used as a cognitive aid and increased speech confidence.	Not reported	Facilitates environment control, organization aid, increased engagement with technology, quality of life, speed of task, accessibility, confidence, reduced frustration and stigma and maintained social contacts.	Opportunities and chances to communicate, entertainment features contribute to motivation, removes social barriers (anxiety and embarrassment), avoids stigma with bespoke support tools and improved quality of life
Disadvantages of VAT	ASR algorithms can smooth over stammering events, frustration with error recovery and unsuccessful strategies and difficulties embedding VAT into daily routines.	Poor recognition of dysarthric speech, frustration and decreased confidence in speech, privacy and security concerns.	Difficulties recognizing mild and profound dysarthric speech may limit clinical applications, and inappropriate as a monitoring tool.	Recognition impacted by fatigue and fluctuations and difficulties impact self‐esteem and confidence, privacy concerns and older adults perceived to be resistant to using technology.	Frustration, privacy issues, continually improving technology impacts therapeutic use, requires ability to set thresholds of recognition at acceptable levels to promote changes in speech intelligibility.

### 3.2 Narrative Review of Findings

Five studies (Bleakley et al. 2022; Smith et al. 2021; Duffy et al. 2021; Kulkarni et al. 2022; Gutz et al. 2022) met the criteria to be included in the review. They were conducted across the United Kingdom (*n* = 3), Ireland (*n* = 1) and the United States (*n* = 1) using a range of methodologies including online mixed methods surveys (*n* = 2), semi‐randomised control trials (*n* = 1), semi‐structured interviews and diary entries (*n* = 1) and non‐randomised experimental design (*n* = 1). Authors reported their primary research targeted people with dysarthria (*n* = 362), other speech difficulties (*n* = 59) or SaLTs (*n* = 261). The total number of participants ranged from 11 (Bleakley et al. 2022) to 290 (Duffy et al. 2021) per study, with an average of 136. This included participants diagnosed with motor neurone disease (*n* = 72) (Gutz et al. 2022), Parkinsons (*n* = 290) (Duffy et al. 2021), intellectual disability (*n* = 48) (Smith et al. 2021), stammering (*n* = 11) (Bleakley et al. 2022) or SaLT's (*n* = 261) (Kulkarni et al. 2022). Populations in the review, with the exception of SLTs, share speech and voice characteristics with Parkinson's Disease, such as reduced clarity or dysfluency.

Duffy et al. (2021) used survey methodology to explore experiences of using VAT and reported effects on speech and voice. Two hundred and ninety PwPD completed the survey, with 166 PwPD reporting a moderate voice impairment on the voice handicap index assessment (VHI), indicating difficulties with volume, clarity and predictability of voice. Within this 166, some indicated that their speech difficulties impacted how well VAT recognised their speech. A number of these participants, 36.7%, reported that they had to sometimes repeat themselves, and 26.5% often had to repeat themselves during VAT interactions. Findings of clinical interest identified that 14.8% of PwPD identified increased clarity of speech, 13% reported increased volume, and 25% indicated they were asked to repeat themselves less after using a commercial smart speaker (VAT). Researchers concluded that the feedback provided by VAT may be a mechanism that promotes positive speech change for people with dysarthria. However, the cross‐sectional nature of the survey meant that it only reflected a single snapshot in time in relation to speech and voice difficulties and interaction with VAT. Duration of VAT use or dysarthria progression was not captured.

Smith et al. (2021) examined the use of two VAT technologies (Amazon Alexa, Google Home) to improve speech intelligibility ratings in 48 adults with intellectual disability, using a semi‐randomised control trial. They found a statistically significant improvement in intelligibility ratings for the intervention group for recordings related to device use, and a slightly smaller effect for unrelated words. Duration of usage ranged from 8 to 20 weeks, with a 12‐week average, but no correlation between duration and intelligibility change was found. Researchers indicated that autonomous, self‐motivated and naturalistic conditions are effective for motivating clients to complete SLT‐style interventions at home, with spaced repetition and device feedback acting in the same capacity as a SLT to affect speech changes. However, given that this was a naturalistic study, it is difficult to measure or assess the impact of dose‐dependent effects on changes in intelligibility. This is the first study to demonstrate an experimental therapeutic effect between VAT and improved speech.

Bleakley et al. (2022) employed diary recordings over 22 days followed by semi‐structured interviews to illuminate the experiences of 11 participants interacting with a VAT, with a stammer. They reported that stammering increased as phrase or sentence length increased. Fatigue and stress also contributed to increased stammering and reduced recognition rate. Participants reported using several speech adaptation strategies to increase recognition rate, including repetition, planning utterances, over‐articulation, speaking slowly, increasing volume and diaphragmatic breathing. Participants also generated fresh findings regarding the potential for VAT to support the therapeutic aims of SaLTs, including the home practice of strategies and unjudgmental feedback. Despite this, device feedback was cited as a source of frustration for many participants, particularly devices timing out, the lack of scaffolding for error repair and the lack of contextual awareness of speech difficulties. Authors concluded that whilst smart speakers hold therapeutic potential, they must be used carefully to avoid negative side effects.

Gutz et al. (2022) used an experimental design to compare ASR, using VAT, with recognition by SaLTs, in terms of severity, intelligibility and sentence length. Findings report that as dysarthria intelligibility decreases, both SaLT and ASR recognition rate decreases. Furthermore, the ASR recognition rate decreases dramatically from normal speech to profound dysarthria. Longer sentences also resulted in lower human and ASR recognition rates and decreased most for the most severe groups with longer sentences. While SaLTs classified dysarthric speech into four severity levels, ASR classified dysarthria as follows: normal, severe and profound, neglecting mild and moderate. Notably, neither SaLTs nor ASR correctly classified moderately dysarthric speech. Although ASR works for broad detection of severe dysarthria, it has limited utility for mild or profound dysarthria. Gutz et al. (2022) concluded that, as ASR had a lower accuracy, sensitivity and specificity range in comparison to SaLTs, poorest performance and a floor effect with increased severities, it was not recommended for monitoring changes in dysarthric speech or in outcome measurement.

Kulkarni et al. (2022) explored SaLTs’ facilitators, barriers and experiences of using VAT in clinical practice for various caseloads. SaLTs reported that low volume, reduced intelligibility and accents were barriers to VAT recognition, and clinicians felt that clients with severe speech impairments would be unable to use VAT successfully. Eleven percent (20/181) did not use VAT as they did not think it would have any therapeutic benefit. Despite this, SaLTs who had used VAT had high success rates with clients with severe speech difficulties. The most clinically relevant finding is that 52.5% of SaLTs believed that VAT could help clients speak louder, and 47.4% believed that it could help clarity of speech. Clinicians who had used VAT reported increased intelligibility, clarity and the VAT's potential to maintain voice in neuro‐degenerative conditions. 7.8% of speech therapists had specifically used VAT where clients had dysarthria, with 55% using the technology to support training on strategies to maintain speech and voice. Other reported uses include VAT as an outcome measurement tool and to support home‐based self‐management practices. Researchers concluded that VAT held potential as a therapeutic tool for clients with a communication impairment and as a clinical tool for SaLTs. However, given that the survey was self‐selective, it may reflect attitudes of clinicians who are more readily embracing technology, positively biasing results.

## 4 Discussion

This review was successful in identifying and mapping the available literature on the utility of VAT for dysarthric speech and speech difficulties. As this is an emerging field, only five studies meeting the inclusion criteria were identified. We identified some recognition rates of VAT, associated with severity and intelligibility for users with dysarthria or a speech difficulty, and impacts on intelligibility, clarity and volume.

There was evidence that VAT has been used with a range of populations experiencing speech and voice challenges, including Parkinson's Disease, motor neurone disease, stammering and intellectual disability. These populations all share similar speech and voice characteristics with Parkinson's Disease, for example, reduced clarity of speech akin to dysarthria or dysfluency similar to palilalia or neurogenic stammering. No literature was identified on the use of VAT for speech assessment; however, literature did indicate a potential role in supporting functional changes and improvements in intelligibility, clarity and volume. Considerations for VAT, potential outcomes and future research are discussed.

### 4.1 Application to Speech Improvement

Our review builds upon a recent report that there is a gap in knowledge regarding the therapeutic potential of VAT for the management of dysarthria and speech difficulties (Cave and Bloch 2023).

#### 4.1.1 Current Usage of VAT

Whilst all five papers in the review had a therapeutic focus, only three papers (Duffy et al. 2021; Smith et al. 2021; Kulkarni et al. 2022) identified therapeutic rationale for use of VAT, including device feedback and support of home practice (Duffy et al. 2021; Smith et al. 2021; Kulkarni et al. 2022). Some SaLTs reported that this influenced their use of VAT in practice (Kulkarni et al. 2022). For example, the technology provides opportunities for unlimited practice attempts. From reviewing the evidence, it is evident that VAT can prompt participants to adapt their speech.

VAT feedback may work in a similar way to LOUD cues provided by SaLTs during LSVT LOUD, improving self‐awareness of speech difficulties and recalibration of volume; a common goal for people with Parkinson's Disease (De Keyser et al. 2016). For example, low volume may result in feedback of misunderstanding or may activate whisper mode on the smart speaker, where the device whispers back to the user. This develops the rationale for VAT usage by people with dysarthria and other speech impairments. VAT provides objective biofeedback on speech clarity and volume, which may contribute to increased self‐awareness and self‐monitoring. However, VAT does provide tailored, person‐centred feedback to cue loudness and re‐train calibration in the same way as LSVT LOUD. This personalised feedback provided by a clinician helps to shape healthy and improved loudness for PwPD, which is not yet possible with VAT. Table 4 shows a comparison between LSVT LOUD and VAT using heuristic indicators informed by this literature review. Notably, our scoping review did not explore the benefits of feedback, and future research should examine the role of such feedback on recalibration.

**TABLE 4:** Showing a comparison between LSVT LOUD and VAT as a therapeutic intervention and therapeutic tool.

Feature	LSVT LOUD	VAT
Effectiveness for clinical outcomes	Significant improvements in vocal loudness, articulation and intelligibility supported by a large evidence base (Ramig et al. 2018) Specifically targets vocal loudness and calibration of internal monitoring with a standardised protocol (Ramig et al. 2018)	Emerging self‐reported evidence of improved volume, clarity and intelligibility of speech; less clinically validated (Duffy et al. 2021; Kulkarni et al. 2022) Can be used to target loudness, intelligibility and functional phrases (Kulkarni et al. 2022; Mills et al. 2025a), but no therapeutic protocol is available.
Feedback	Real‐time feedback from a human therapist, used to cue and shape vocal loudness	Limited to basic device feedback, for example, ‘Sorry I didn't understand that’ or no feedback at all (Mills et al. 2025a)
Dosage	Highly structured and intensive: Four sessions a week for 4 weeks (Ramig et al. 2018)	Unstructured personal usage, depending on installed skills; variable (Mills et al. 2025b). No recommendations for dosage
Personalisation	Therapy tailored by a trained therapist to the client's goals (Ramig et al. 2018)	Therapy customisation is not available; however may be used to practice some salient materials (Mills et al. 2025a)
Accessibility	Requires a trained therapist, which can be difficult to access in the United Kingdom (Miller et al. 2011) or expensive	Readily available at home, relatively low cost, no scheduling needed (Mills et al. 2025b)
Convenience	Requires available appointments, trained clinicians and travel	Can be used anytime at home, passively or casually
Practice motivation	High effort exercises may lead to practice abandonment and poor self‐reported maintenance of therapy gains for PwPD (Yorkston et al. 2017; Baylor et al. 2023), but this may be mitigated by continued SaLT input and feedback.	Allows everyday functional practice, which may be motivating (Smith et al. 2021). Lack of interaction realism may result in the novelty of technology wearing off, but it may be used as a practice tool for some exercises in conjunction with LSVT LOUD (Mills et al. 2025a; Mills et al. 2025b).

Additionally, three papers suggested that VAT could support home‐based therapy programmes, practice and self‐management. Indeed, participants with a stammer in one study directly suggested the use of VAT for SLT. It is tentatively suggested that VAT may allow easy, frequent use in an everyday environment, which indirectly contributes to reinforcement and continued motivation to use the device at home. VAT's everyday nature reduces stigma, which may be traditionally associated with specialist SLT equipment (Kulkarni et al. 2022), contributing towards practice motivation and integration of practice into daily routines. This may overcome some reported limitations with therapies like LSVT LOUD, where the high effort exercises may lead to practice abandonment and poor self‐reported maintenance of therapy gains for PwPD (Yorkston et al. 2017; Baylor et al. 2023). However, LSVT LOUD remains the gold standard therapeutic intervention for PwPD, where high effort exercises generate significant improvements in volume and intelligibility (Ramig et al. 2018). While PwPD may have an initial motivation to practice with VAT, the lack of interaction realism may result in the novelty of the technology wearing off. Continued SaLT input and feedback may help to motivate and challenge PwPD to continue progressing, whilst the convenience and accessibility of VAT aid practice motivation.

Despite these findings, wider literature indicates that ASR models used in devices and the utterance complexity of speech tasks may impact device accuracy (Wiepert et al. 2023). Cave (2024) has suggested that ASR errors are higher than expected for dysarthric speech, and the ASR model often did not recognise words participants used, such as proper nouns, names and locations. This indicates that it may be difficult to use VAT to practice personalised phrases, beyond basic interactional requests.

Furthermore, ethnicity, accent and geographical background have also been shown to affect ASR performance and may affect unjudgmental feedback. Accent and varied pronunciation of individuals speaking a second language may increase the word error rate (WER) of ASR (Radzikowski et al. 2021). A higher average WER has been reported for black speakers than white when used with common commercial ASR systems like Apple, Amazon and Google. Similarly, the WER of an English‐trained ASR was higher for Scottish speakers than those from America or New Zealand (Koenecke et al. 2020; Tatman and Kasten 2017). As such, VAT may present an ethnocentric and language bias towards English speakers. This suggests that while VAT users may change volume and clarity of speech to interact with VAT, they may also change non‐standard accents to more westernised accents to facilitate interactions with ASR (Mengesha et al. 2021). This is likely to have implications for users with speech and voice difficulties who have regional accents or dialects or come from an ethnic minority background. Therefore, SaLT's should evaluate the following factors when considering the use of VAT, given that VAT provides little feedback on why speech may or may not be recognised: (1) Does speech recognition by VAT reflect how well speech is recognised in everyday life? (2) Can you pinpoint if the error is device or speech‐related? (3) Is using VAT causing undue frustration or psychological harm to the user? (4) Has there been a lack of progress towards goals since starting to use VAT?

Without clear and specific feedback on device or speech errors, clinicians are left without the critical information needed to tailor therapy effectively to individual needs. This may undermine SaLT's ability to set appropriate therapy goals, monitor meaningful progress or provide targeted feedback. Worse still, it risks reinforcing maladaptive speech behaviours if clients adjust their speech based on inconsistent or misleading responses from the device. Such unintended consequences not only compromise therapeutic efficacy but may also damage client motivation and confidence. Therefore, highlighting the risks associated with this lack of transparency is essential to ensure that VAT is integrated into clinical practice in a way that is both safe and evidence‐informed. Therefore, clinicians should carefully consider the risk–benefit analysis of using VAT with their clients, on a case‐by‐case basis. Table 5 presents additional risks associated with VAT usage and should be thoroughly considered by clinicians seeking to implement VAT in clinical practice.

**TABLE 5:** Showing advantages and limitations that may occur when using VAT, as retrieved from the reviewed literature. This is mapped to the International Classification of Functioning (ICF), showing voice‐related impacts of VAT use on activity, participation and well‐being (World Health Organisation (WHO) 2001).

	Self‐reported advantage of VAT	Self‐reported limitation of VAT (potential adverse effects)
Impairment	Speech overcomes barriers associated with co‐morbid difficulties, for example, difficulties with typing due to tremor (Duffy et al. 2021) Improved volume (Duffy et al. 2021) Improved articulation (Duffy et al. 2021, Smith et al. 2021) Improved clarity (Duffy et al. 2021)	Can be unreliable with disease fluctuations, for example, on/off periods (Gutz et al. 2022) Poor recognition of dysarthric speech—Exclusionary to people with speech difficulties (Gutz et al. 2022, Kulkarni et al. 2022) Incorrect reinforcement of speech production (Kulkarni et al. 2022)
Activity	Increased engagement with technology (Duffy et al. 2021) Increased speed of task (Kulkarni et al. 2022) Increased speech and voice‐related independence (Kulkarni et al. 2022) Provides opportunities/chances for communication (Smith et al. 2021) Entertainment features contribute to interaction motivations (Smith et al. 2021)	
Participation		Communication avoidance—Due to increased awareness of difficulties (Bleakley et al. 2022)
Personal (well‐being)	Increased confidence (Duffy et al. 2021, Kulkarni et al. 2022) Increased speech confidence (Duffy et al. 2021)	Frustration with recognition and error recovery (Duffy et al. 2021; Bleakley et al. 2022) Decreased confidence (Duffy et al. 2021) Negative impacts on self‐esteem and self‐perception (Bleakley et al. 2022) Reduced comfort and ease with smart speakers (Bleakley et al. 2022)
Environment	Removes social barriers—Anxiety and embarrassment from having to repeat to people (Duffy et al. 2021; Bleakley et al. 2022) Lack of perceived judgement (Duffy et al. 2021; Bleakley et al. 2022, Kulkarni et al. 2022) Avoids stigma associated with bespoke communication tools (Duffy et al. 2021; Kulkarni et al. 2022) Increased motivation to complete therapy (Kulkarni et al. 2022) Controlled speaking environment—Reduced anxiety (Bleakley et al. 2022)	Difficulties embedding VAT into daily routines, for example, proximity, presence of others (Bleakley et al. 2022) Older adults perceived to be unwilling and resistant to using technology (Duffy et al. 2021, Kulkarni et al. 2022) Privacy and security concerns (Duffy et al. 2021, Kulkarni et al. 2022) May be impacted by background noise (Bleakley et al. 2022)

Research has suggested leveraging technology to maintain therapy gains for PwPD (Swales et al. 2020), and VAT may be a potential candidate. Indeed, research has used technology at home following LSVT LOUD, known as Companion, and demonstrated gains in volume. Although gains were comparable with standard in‐person LSVT LOUD (Halpern et al. 2012), desktop technology varies greatly from VAT, making outcome generalisation difficult. This evidence mapping has indicated the potential of VAT; however, the low number and low quality of studies mean it is not possible to determine how it could complement SLT or maintain speech following discharge. As previously discussed, the convenience and accessibility of VAT indicate it holds potential as a practice tool to support the practice of some LSVT LOUD exercises (Table 4).

Despite the various therapeutic benefits of VAT (Duffy et al. 2021; Smith et al. 2021; Kulkarni et al. 2022), one of the papers (Kulkarni et al. 2022) indicates that the majority of SaLTs are not yet using it therapeutically. There may be several reasons for this, including a lack of agreed intervention protocols, insufficient evidence and a lack of practice guidelines. For example, there is limited research underpinning how VAT use drives speech changes or guides the dosage of intervention use. Research has suggested that immediate objective feedback from VAT, spaced repetition, the engaging and motivational nature of VAT, removal of social stigma and VAT acting as a conversation starter with others contribute to VAT's mechanism of change (Smith et al. 2021; Makin et al. 2025). However, only one study (Makin et al. 2025) has begun to establish the impact of these variables on speech change, in a paediatric population, indicating that additional research is needed. Furthermore, it is possible that the impact of VAT may be purely attributed to an increase in the use of voice. Given that Parkinson's Disease has different underpinning neurological changes which affect feedback loops, it is suggested that the mechanism of effect with this population may be different.

Furthermore, only one report gave the intervention duration, which averaged at 12 weeks, ranging between 8 and 20 weeks. Therefore, there is a gap in the evidence base regarding how VAT drives speech changes for PwPD, the therapeutic use of VAT and practical implementation. These gaps in knowledge may contribute to reduced perceptions of clinical usefulness and partially explain reduced adoption of smart speakers by SaLTs. Further research is required to establish the means by which smart speakers improve volume and intelligibility in adults (Smith et al. 2021; Makin et al. 2025) and establish dosage recommendations for clinical usage. However, it is acknowledged that people with speech and voice difficulties may not primarily use VAT for speech practice, but rather because it is a useful and functional tool in their lives. This suggests that improvements in speech intelligibility or voice may be a secondary outcome of usage, rather than the main factor for adoption of VAT (Smith et al. 2021).

#### 4.1.2 Speech and Voice Outcomes

VAT may improve outcomes for intelligibility, clarity and volume of speech (Duffy et al. 2021; Smith et al. 2021). Reduced repetition, reduced rate of speech and improved self‐awareness of speech are also reported, with diaphragmatic breathing and planning utterances mentioned as facilitative strategies to enable clearer speech. Wider literature concurs (Denman and Jones 2019; Makin et al. 2025), where people with voice difficulties reported speaking slowly and loudly when interacting with Amazon Alexa (Pradhan et al. 2018). Additional research focussing on the use of Internet of Things technology for PwPD indicates that they could improve recognition when they modified their speech to interact with Alexa (McNaney et al. 2020). Moreover, validation of these improvements relies on potentially biased self‐reported measures. This limitation has been acknowledged, with a clear need to strengthen the evidence base, examine outcomes for specific clinical cohorts and assess functional benefits.

Challenges with VAT recognition result in people modifying their speech and indicate VAT's therapeutic potential to promote clinically meaningful speech changes for people with speech difficulties (Smith et al. 2021). More severe speech difficulties may have lower recognition, requiring the use of additional adaptive speaking strategies. However, it should be noted that not all speech and voice difficulties may be open to remediation when using VAT. For example, dysfluency, palilalia or vocal tremors may not cease following VAT use, but may negatively impact recognition of speech (Bleakley et al. 2022). A lack of evidence in this area makes it difficult to draw firm conclusions. Additionally, there is a lack of evidence regarding how nasality, intonation or pitch changes as a result of dysarthria may be impacted therapeutically by the use of VAT.

Given that only one paper in this review (Smith et al. 2021) used pre‐ and post‐measures to explore changes in speech outcomes following VAT use, findings should be interpreted cautiously. There is a pressing need for future research to quantitatively examine if VAT can improve volume and intelligibility of speech and establish a firmer evidence base. This may replicate Smith et al. (2021) and Makin et al. (2025) with an adult population, such as Parkinson's Disease. However, the professional and user perspectives of VAT usage reported here and in wider literature (Mills et al. 2025a; Mills et al. 2025b) positively contribute to the foundations of evidence‐based practice. As the area is novel with technology rapidly developing, there is currently no common methodology noted when measuring or evaluating speech and voice outcomes using VAT.

#### 4.1.3 Functional Gains

This review cautiously suggests that VAT may hold the potential to impact speech and voice‐related impairment, activity, participation and well‐being, and maps this to the ICF categories (Table 5). However, it is important to note that no studies directly investigate if the therapeutic use of VAT for speech and voice difficulties also improves activity, participation and well‐being. Impacts on activity, participation and well‐being are related to the use of VAT as a rehabilitation tool for speech and voice, rather than as an accessibility tool for PwPD.

Four studies (Duffy et al. 2021; Smith et al. 2021; Kulkarni et al. 2022; Bleakley et al. 2022) consistently report subjective improvements in confidence, self‐awareness and well‐being when using VAT for speech and voice difficulties. This concurs with wider literature demonstrating generally increased independence, well‐being and quality of life in older adults and people with disabilities (Werner et al. 2023). The self‐reported speech and voice‐related impacts below (Table 5) may provide a starting point for exploration.

Decreased confidence, frustration with increased repetition, security anxieties and avoidance of communication with smart speakers due to increased awareness of speech difficulties were also noted. It is difficult to know if improvements in intelligibility, clarity or volume following VAT use could be outweighed by negative emotional consequences. Cave (2024) concluded that clinicians and service users should be mindful that ASR models can be variable in their understanding of speech, for several reasons beyond speech clarity, volume and intelligibility, as discussed above. In the paper, a person with ALS blamed herself whenever the ASR tool did not caption her words correctly. This highlights potential negative implications for well‐being, which can arise from VAT use. Therefore, clinicians should be careful not to assign responsibility for all VAT inaccuracies to speech and voice and should use their own clinical judgement to carefully consider which clients are suitable for VAT use, on a case‐by‐case basis.

Furthermore, speakers can link ASR errors to their own sense of identity, including racial, regional and location identity, and participants may modify their dialect and word choice in order to be understood by ASR (Mengesha et al. 2021). Although the papers in the review focus on English speakers, this is a concern for dysarthric users who reside outside of the United States and United Kingdom and speak a foreign language (Ayoka et al. 2024), given that VAT recognition rates may be even lower. Therefore, it is a concern that the use of VAT for SLT may not enable rehabilitation and communication equity in global countries, especially where speakers of minority languages are often part of marginalised groups (Ayoka et al. 2024). Future research is needed to establish the psychosocial impacts of using VAT for SLT for dysarthric users who speak a foreign language and compare recognition rates across English and foreign languages. Therefore, clinicians should exercise clinical judgment when using VAT and cease use if a change occurs beyond adaptive speaking strategies. It is essential that SaLTs mitigate this risk by educating potential VAT users on strategies for adapting speech, and about the lack of training of ASR on dysarthric speech and some minority, foreign languages. Further adverse effects associated with VAT usage are reported in Table 5 and should be thoroughly considered by clinicians seeking to implement VAT in clinical practice.

Additionally, security and General Data Protection Regulation (GDPR) concerns are also reported across the literature and are considered a barrier where VAT is used in a healthcare setting (Werner et al. 2023). Therefore, future research should consider how VAT could be used to improve intelligibility and volume of speech in line with national legal and governance requirements.

#### 4.1.4 Populations Studied

Searches returned evidence of four clinical populations with sample sizes ranging between 11 and 290. This creates difficulties generalising the results to other user groups or selecting the optimal population for VAT use. This is due to a lack of available evidence in the area. Previous research has also emphasised a gap in knowledge regarding the potential for VAT as a tool for supporting people with ALS, a neuro‐degenerative condition (Cave and Bloch 2023). In the context of the above findings, we conclude that this warrants further research into the therapeutic potential of VAT for neuro‐degenerative conditions, as a tool for speech and voice difficulties. Furthermore, given that all populations included in the review share speech and voice difficulties with those observed in Parkinson's Disease, it may be a potential population for application. For example, reduced clarity and intelligibility in dysarthria associated with intellectual disability and dysfluency, which is a common feature in Parkinsonian speech. Two papers (Duffy et al. 2021; Smith et al. 2021) recommend that additional research explores the use of VAT for people with Parkinson's; however, no identified research has included PwPD as end users in the development of a VAT intervention.

Additionally, a lack of research fitting our inclusion criteria means that it is difficult to understand how severities of speech and voice difficulties interact with VAT (see below). Although SaLTs often prioritize impairment‐based interventions for earlier stage conditions (Collis and Bloch 2012), without research investigating the interaction of mild, moderate and severe speech difficulties with VAT, it is difficult to draw a firm conclusion on a user group.

#### 4.1.5 Other Considerations in a Therapeutic Context

There may be other considerations for the therapeutic usage of VAT in SLT, such as how well speech is recognised; however, varying methodologies between studies (Duffy et al. 2021; Bleakley et al. 2022; Gutz et al. 2022) hinder firm conclusions on how well VAT recognizes dysarthria and speech difficulties.

People with Parkinson's report varied recognition rates: 43.4% find it effective most of the time (Duffy et al. 2021). As with two other papers (Gutz et al. 2022; Bleakley et al. 2022), this suggests that VAT recognises mild or moderately dysarthric speech sufficiently well. This trend aligns with broader ASR literature (Young and Mihailidis 2010; Mustafa et al. 2015; Jaddoh et al. 2023), where recognition rates decrease with reduced intelligibility and increased severity of dysarthria. When mild or moderate dysarthria is well recognised by VAT, this is problematic in SLT, as the same speech may not be recognised in functional settings by listeners. Therefore, this may problematise the use of VAT as a measure of intelligibility in the clinic, as it may not reflect the challenges of real‐life communication difficulties. Improved recognition of mild and moderate dysarthria may be attributed to projects like Google Euphonia, which trains VAT on dysarthric speech, enhancing recognition and accessibility. While positive for VAT's development and user confidence, this insensitivity to disordered speech complexities warrants further research regarding VAT's role in SLT as the technology continues to evolve and improve.

We found no longitudinal research examining changes in speech recognition rates across progressive conditions, ranging from mild, moderate, severe or profound difficulties. This is pertinent as SaLTs have different therapy focusses for mild, moderate and severe dysarthria (Collis and Bloch 2012), and it is difficult to know how VAT clinical utility may differ at different stages of disease progression. Future research is needed to examine WER in conditions like Parkinson's in relation to SLT assessment, management and outcome measurement.

Furthermore, two of the reviewed papers, which discussed recognition, did not directly investigate how severity, intelligibility or speech difficulties affect recognition outcomes such as WER (Duffy et al. 2021; Gutz et al. 2022). Some papers recognize this limitation, attributing it to GDPR and the inability to access participant speech recordings. Transcriptions of sentences are necessary for a complete understanding of recognition and error rates, which is difficult in clinical practice due to ethical and data privacy concerns, potentially limiting usage.

SaLTs often seek to understand the impact of speech and voice impairments on conversational understanding and clients’ ability to accurately convey meaning. While VAT can provide feedback on intelligibility, the transactional nature of requests does not reflect the functional nature of conversations, where meaning can be conveyed through body language, gesture and contextual understanding (Cave 2024). Consequently, conversational interaction with VAT may not be reflective of functional conversation for PwPD and their communication partners (Cave 2024). Therefore, interactions with VAT may not account for compensatory strategies used during communication and may not provide a holistic picture of the user and the impacts of their impairment on everyday functioning. These subtleties make the case for cautious implementation of VAT as a clinical tool, informed by clinical judgement.

Additionally, research demonstrates that VAT poorly recognises conversational speech by people with dysarthria (Tobin et al. 2024), and recognition errors for conversational speech are significantly higher than short, read phrases or scripted, read speech (Green et al. 2021; Gabler et al. 2023). Given that recognition errors are high for conversational dysarthric speech, it would be difficult for clinicians to establish whether errors are device‐based or speech‐based. Future research should explore and compare VAT recognition rates for words, sentences, phrases and conversational speech. This may allow clinicians to establish where on the speech hierarchy VAT is best placed to facilitate practice.

To summarise, recognition rates are currently unreliable. As some VAT devices recognise mild and moderate speech well, this also means that they may gloss over or ignore mild or moderate speech impairments. Furthermore, VAT is unreflective of functional communication assessment at the conversational level. Notably, no papers included in the review have used VAT as an assessment tool for dysarthria or speech difficulties in a speech and language assessment content. For example, grading the severity of dysarthria or the perceptual characteristics of speech. This reflects the fact that VAT is a commercial technology and not solely designed for a therapeutic purpose. Whilst some papers in the review report VAT's recognition rate of dysarthric speech, this cannot be extrapolated to be indicative of how VAT may perform as a screening tool or to assess intelligibility and volume in a SLT context.

### 4.2 Summary

VAT has potential applications for the management of speech and voice disorders. Despite a lack of quantifiable evidence, it may be used to promote useful changes in volume, intelligibility and clarity. The available evidence is far from complete. There is a need for quantitative measurement of the effect of VAT usage on volume and intelligibility. Furthermore, it remains unclear if and how the therapeutic use of VAT may impact activity, participation and well‐being. Based upon our findings, further work is needed to secure the place of VAT in SLT, whether as an outcome measurement tool for speech changes, or to support home practice pre‐ or post‐formal SLT intervention.

Several barriers to VAT implementation have been identified in considering outcomes in relation to risks and adverse effects of VAT. For example, we note high WER for conversational dysarthric speech, ethnocentric bias and potential impacts on well‐being and identity. Additionally, the reliability and suitability of VAT as an assessment or screening tool remains unknown. There is no evidence to apply the usage of VAT to assessment in SLT, and further research exploring if VAT holds utility for assessment of conversational speech may be warranted. Future research may also establish how VAT recognises different severities of speech and the consequential impact for improvements in speech intelligibility, clarity and volume.

Overall, clinicians should use their own clinical judgement to carefully consider the risk–benefit analysis of using VAT with their clients, on a case‐by‐case basis.

## 5 Limitations

Selection bias is a possibility in the review, as two of the co‐authors (O.D. and W.G.K.) were involved in two included studies. However, all studies were assessed for inclusion by two independent reviewers, disagreements were resolved by consensus discussion and involved authors did not complete data extraction from their own papers. These measures should have mitigated risk.

Only papers available in English were included in the review. Given that VAT has different recognition rates for foreign languages (Hassan and Chiu 2022), this may limit the applicability of VAT in SLT for speakers of languages other than English.

Additionally, the focus on VAT, which is a continually evolving topic, means findings may quickly become outdated if recognition technology is improved for dysarthric speech and speech difficulties. ASR models in VAT can change without warning with patches and updates, presenting a risk of change in baseline measurements and for the attainability and measurement of therapy goals.

Furthermore, projects such as Voiceitt, Google Euphonia and Project Relate aim to improve ASR accuracy in recognizing dysarthric speech, which may have the unintended consequence of limiting certain therapeutic applications of VAT. However, these tools are still unlikely to be accurate and have their own limitations that must also be solved. Future VAT designs should allow users to set recognition rates to enable both increased accessibility and clinical usage. Additionally, given the wealth of literature emerging in this particular area, it is likely that additional literature may have emerged during the time from submission to publication.

## 6 Implications for Research

To better understand how VAT can be used by SaLTs and people with dysarthria to promote speech and voice changes and maintenance, the following research areas have been proposed (based upon synthesis of findings from our review):

- To explore SaLTs, carers and PwPD's experiences of using VAT with a speech or voice difficulty, to understand facilitators and barriers to use, therapeutic uses of VAT and how unmet needs can be addressed.
- To create a co‐designed intervention with SaLTs, PwPD and carers, using VAT to help speech and voice difficulties.
- To quantitatively test the effect of VAT usage on volume and intelligibility for adults. This may inform an intervention protocol for VAT usage with dysarthric speakers. For example, dosage, duration and context of use, type and nature of requests, distance from speaker and presence of background noise.
- To examine how VAT can improve speech outcomes using a pre–post design with standardized SLT measures and/or acoustic analysis and to explore how long this is maintained following the intervention.
- To investigate if the therapeutic use of VAT for speech and voice difficulties can also lead to participation and well‐being changes, and how these act as facilitators and barriers to usage.
- To explore the use of VAT with neuro‐degenerative populations with speech and voice difficulties. Research should ascertain how VAT recognises mild, moderate and severe speech impairments and how this impacts therapeutic improvement across the progression of neuro‐degenerative disease.

## 7 Conclusion

Findings offer a starting point for further exploration of VAT by people with dysarthria or speech difficulties, specifically, PwPD and SaLT's involved in their management. There is limited quantifiable evidence to suggest routine use of VAT to promote improved volume, clarity and intelligibility of speech. Feedback, and home practice support, may facilitate increased self‐awareness and foster self‐management of dysarthria or speech and voice difficulties; however, error rates and potential impacts on well‐being must be weighed up. Next steps include qualitative work with SaLTs, PwPD and carers to explore experiences using VAT with speech and voice difficulties.

## Ethics Statement

The authors have nothing to report.

## Consent

The authors have nothing to report.

## Conflicts of Interest

The authors declare no conflicts of interest. The review presented here was completed in partial fulfilment of Jodie Mills’ PhD at Ulster University.

## Permission to Reproduce Material for Other Sources

Papers included in the review were Open Access. We did not use any copyrighted questionnaires.

## Supporting information




**Supporting File 1**: jlcd70140‐sup‐0001‐SuppMat.docx

**Supporting File 2**: jlcd70140‐sup‐0002‐Checklist.docx

## Data Availability

The data that support the findings of this study are available from the corresponding author upon reasonable request.
