# Supplementary material for: Voice‐Assisted Technology as a Potential Tool for Addressing Speech and Voice Concerns Experienced by People With Parkinson's Disease and Other Conditions Presenting With Dysarthria: A Scoping Review
Source: Int J Lang Commun Disord. 2025 Oct 20;60(6):e70140. doi: 10.1111/1460-6984.70140 (PMC12537997; doi:10.1111/1460-6984.70140)
Supplement: Supplementary file 1 — Supporting File 1: jlcd70140‐sup‐0001‐SuppMat.docx [file JLCD-60-0-s002.docx]

**Supplementary Materials for Review**

**Supplementary Material 1 – Search Strategy**

| dysarth* OR "speech impair*" OR "speech intelligibility" OR (voice or communicat* OR vocal or articulat* or impair*) N2 (disorder* or difficult* or problem*) | **AND** | "voice assisted technolog*" OR "smart speaker" OR alexa or siri or "google assistant" or cortana or bixby OR "voice integrated technology" or "voice user interface" or ASR or "automat* speech recognition" or "speech recognition technology" or "voice interaction" OR (google or digital or online or voice or virtual or speech or "intelligent personal") n2 (assistant or technolog*) | **AND** | intelligibility OR "speech difficult*" or frequency or volume or clarity or loudness or voice OR speech N2 (improv* or gain*) |
| --- | --- | --- | --- | --- |

**Supplementary Material 2 – Data Extraction and charting framework**

| Title / Journal Info |  | Finding |
| --- | --- | --- |
| Country of Origin |  |  |
| Aims and Objectives |  |  |
| Overview of study characteristics | Design |  |
|  | Procedure |  |
|  | Type of data collected |  |
| Participant Demographics (gender, age, diagnosis) |  |  |
| Sample Size |  |  |
| Inclusion / Exclusion criteria |  |  |
| Time since diagnosis / disease severity |  |  |
| Participant communication needs |  |  |
| Technology used |  |  |
| Execution of delivery |  |  |
| Dosage and duration of intervention |  |  |
| Exposure |  |  |
| Purpose of VAT use |  |  |
| Findings – recognition outcomes | How well is dysarthric speech recognised? |  |
|  | Impact of utterance length |  |
|  | What speech characteristics impact recognition? |  |
| Findings – speech changes | Direct speech changes |  |
|  | Strategies to adapt speech |  |
|  | How could VAT be used as a therapeutic tool for SLTs? |  |
|  | Advantages of VAT |  |
|  | Limitations of VAT |  |
| Researcher reported limitations |  |  |
| Implications for future research |  |  |
